# Transvaginal Sonographic Evaluation of the Cervix in Asymptomatic Singleton Pregnancy and Management Options in Short Cervix

**DOI:** 10.1155/2012/201628

**Published:** 2012-02-22

**Authors:** Resul Arisoy, Murat Yayla

**Affiliations:** ^1^Department of Obstetrics and Gynaecology, Gole State Hospital, 34660 Ardahan, Turkey; ^2^Department of Perinatology, Obstetrics and Gynecology, International Hospital, 34660 Istanbul, Turkey

## Abstract

Preterm delivery (PTD), defined as birth before 37 completed weeks of gestation, is the leading cause of perinatal morbidity and mortality. Evaluation of the cervical morphology and biometry with transvaginal ultrasonography at 16–24 weeks of gestation is a useful tool to predict the risk of preterm birth in low- and high-risk singleton pregnancies. For instance, a sonographic cervical length (CL) > 30 mm and present cervical gland area have a 96-97% negative predictive value for preterm delivery at <37 weeks. Available evidence supports the use of progesterone to women with cervical length ≤25 mm, irrespective of other risk factors. In women with prior spontaneous PTD with asymptomatic cervical shortening (CL ≤ 25 mm), prophylactic cerclage procedure must be performed and weekly to every two weeks follow-up is essential. This article reviews the evidence in support of the clinical introduction of transvaginal sonography for both the prediction and management of spontaneous preterm labour.

## 1. Introduction

Preterm delivery occurs in 5–13% of pregnancies before 37 weeks' gestation. Preterm delivery is a major cause of perinatal morbidity and mortality [1–7]. Most of the damage and death cases occur in infants delivered before 34 weeks. The incidence of early preterm delivery (<34 gestational weeks') is 1–3.6% [1, 2]. Preterm delivery is associated with a high prevalence of severe neurological deficits and developmental disabilities and is a leading cause of infant and neonatal mortality. Preterm neonates are at increased risk of developing respiratory distress syndrome, bronchopulmonary dysplasia, sepsis, intraventricular hemorrhage, patent ductus arteriosus, necrotizing enterocolitis, and disorders related to gestational age at birth [8, 9].

Risk factors for preterm delivery include demographic characteristics, behavioral factors, and aspects of obstetric history such as previous preterm birth. Demographic factors for preterm labor include black race, extremes of maternal age (<18 or >35), low socioeconomic status, and low prepregnancy weight. Preterm labor and birth can be associated with stressful life situations (e.g., domestic violence, close family death, work and home environment) either indirectly by associated risk behaviors or directly by mechanisms not completely understood. Many risk factors may manifest in the same gravida [1–3, 10].

The exact mechanism of preterm labor is largely unknown but is believed to include decidual hemorrhage (e.g., abruption, mechanical factors such as uterine overdistension from multiple gestation or polyhydramnios), cervical incompetence (e.g., cone biopsy), müllerian duct abnormalities, fibroid uterus, cervical inflammation (e.g., resulting from bacterial vaginosis, trichomonas), maternal inflammation and fever (e.g., urinary tract infection), hormonal changes (e.g., mediated by maternal or fetal stress), and uteroplacental insufficiency (e.g., hypertension, insulin-dependent diabetes, drug abuse, smoking, alcohol consumption). Each of these underlying causes can initiate the cascade of events that ultimately lead to uterine activity and cervical dilation. Thus, a reduction in the spontaneous PTD rate may require not only accurate identification of patients at risk for preterm delivery but also effective treatment strategies aimed at correcting the underlying causes of preterm labor [1–3, 10–16].

Methods used for predicting preterm birth include risk scoring system, assessments of salivary estriol, fetal fibronectin (FFN), maternal serum alpha fetoprotein (MS-AFP), cervicovaginal intracellular adhesion molecule-1 (ICAM-1), phosphorylated insulin-like growth factor binding protein-1 (phIGFBP-1), cervicovaginal beta-human chorionic gonadotropin (*β*-hCG), and the cervical morphology and biometry. While hospital tocodynamometry has been effective for monitoring uterine contractions to evaluate preterm labor, home uterine activity monitoring (HUAM) has not been proven valuable in detecting or preventing preterm birth and is not currently recommended for use [17, 18].

## 2. Biomarkers of Preterm Birth

The most commonly used and most predictive method for preterm birth is fetal fibronectin. Fetal fibronectin (fFN) is a glycoprotein produced by fetal membranes and trophoblasts which form a biological glue that adheres the fetal membranes and placenta to the decidua. Before approximately 20 gestational weeks it is normally found (4%) in secretions of the cervix and vagina. Thereafter it is a pathological finding and a marker of choriodecidual disruption [19–21]. Initially, Lockwood et al. reported that the presence of cervicovaginal fetal fibronectin in the second and third trimesters of pregnancy identifies a subgroup of women who are at high risk for preterm delivery. They showed that fFN had a sensitivity of 81.7% and specificity of 82.5% for detecting PTD at 37 weeks of gestation in asymptomatic patients [19]. The systematic review by Honest et al. demonstrated that in asymptomatic women the best summary likelihood ratio for positive fFN results was 4.01 (95% confidence interval 2.93 to 5.49) for predicting birth before 34 weeks' gestation, with corresponding summary likelihood ratio for negative fFN results of 0.78 (0.72 to 0.84). Among symptomatic women the best summary likelihood ratio for positive results for fFN was 5.42 (4.36 to 6.74) for predicting birth within 7–10 days of testing, with corresponding ratio for negative fFN results of 0.25 (0.20 to 0.31) [22].

Recently, cervical or vaginal fetal fibronectin is the most powerful biochemical prediction marker of SPTD due to the high negative predictive values [23, 24]. Deplange et al. investigated a sequential test with fetal fibronectin detection after ultrasound measurement of cervical length to predict preterm delivery in women with preterm labor. They reported that the sensitivity, specificity, and positive and negative predictive values of fetal fibronectin positiveness were 75, 71, 17, and 97% for delivery within 14 days; those of cervical length inferior or equal to 20 mm were 75, 52, 21, and 92% for delivery before 34 weeks. The efficiency of the sequential test was similar with excellent negative predictive value: sensitivity, specificity, and positive and negative predictive values of 75, 63, 26, and 93.5% for prediction of preterm delivery before 34 entire weeks. The use of this sequential test could have avoided 37% of fibronectin tests [25]. 

The maternal salivary E3 level seems to correlate well with the serum level and it has been shown that elevated maternal serum E3 levels are associated with increased risk of preterm birth in asymptomatic and symptomatic women presenting for symptoms of preterm labor [26]. It also has low sensitivity and is currently mainly used in clinical settings due to its negative predictive value (i.e., women who test negative are at very low risk of preterm birth and no interventions are necessary) [17, 27]. This test is thus currently more useful for research than for clinical practice [27]. 

Previous research has showed an association with elevated AFP and adverse pregnancy outcomes, including spontaneous preterm birth. The premature delivery screening can be used at the beginning of the 2nd trimester. Cut-off value of 1.8 MoM for marking the higher-risk group was used for marking the high-risk group. Women with equal or higher values of AFP were 3.8 times more likely to have premature delivery than those with lower AFP values (95% CI: 2.2; 6.3). Sensitivity of 25% and specificity of 92% were proven [28]. At a patient-level meta-analysis of 24 studies by Yuan et al., there was no association with preterm birth (OR = 1.80, 95% CI: 0.92–2.68) at women in whom AFP was elevated in isolation. Their findings suggest that maternal AFP levels are strongly related to preterm birth, but only in the context of other abnormal pregnancy markers [29].

Marvin et al. reported that measurement of sICAM-1 in cervicovaginal fluid has potential as a predictor of preterm delivery in women with symptoms of preterm labor. Elevated sICAM-1 concentrations predicted short intervals to delivery (area under receiver operator characteristic (ROC) curves, 0.70–0.72 for delivery within 3, 7, and 10 days), with high specificity. Characteristics for delivery within 3 days at a 3 ng/mL threshold for a positive test were sensitivity of 33.3%, specificity of 98.9%, and positive and negative predictive values of 75.0% and 93.9%, respectively. Predictive ability was independent of and complementary to that of fetal fibronectin [30]. Kwon et al. showed that ICAM-1 gene K469E polymorphism may be a candidate region and useful predictor of susceptibility to PTD [31].

The recent studies showed that a new cervicovaginal test to detect phosphorylated insulin-like growth factor binding protein-1 (phIGFBP-1) may improve the accuracy of predicting preterm delivery. The phIGFBP-1 is mainly secreted by maternal decidual cells and may be an indicator of tissue damage of the choriodecidual interface. In the first trimester, 24.5% of women, and in the mid-second trimester, 20.2% of women, had an elevated cervical fluid phIGFBP-1 level [32, 33]. Rahkonen et al. investigated an assessment of phIGFBP-1 in predicting preterm delivery in 5180 unselected pregnant women. They found that the rates of spontaneous PTD before 32 and before 37 weeks of gestation were higher in women with an elevated cervical fluid phIGFBP-1 level, compared with women who had cervical phIGFBP-1 of <10 micrograms/L (1.1% versus 0.3% and 5.7% versus 3.2%, resp.). An elevated phIGFBP-1 level in the first trimester was an independent predictor for PTD before 32 and before 37 weeks of gestation, with odds ratios of 3.0 (95% CI 1.3–7.0) and 1.6 (95% CI 1.2–2.3), respectively. Cervical phIGFBP-1 levels of 10 micrograms/L or more in the first trimester predicted PTD before 32 and before 37 weeks of gestation, with sensitivities of 53.8% and 37.0%, respectively. The negative predictive values were 99.7% and 96.8%. They showed that elevated cervical fluid phIGFBP-1 levels in the first trimester were associated with an increased risk of spontaneous PTD [34]. 

In another study, Rahkonen et al. investigated that short cervix (<25 mm), positive phIGFBP-1 test, combination of both, and clinician's judgment were all associated with preterm delivery < or = 34 weeks or within 14 days in a total of 246 women between 22 and 34 weeks of gestation. The negative predictive values for delivery < or = 34 weeks were 97.4, 97.6, 97.1, and 98.7%, respectively, and within 14 days 98.7, 99.0, 98.3, and 99.6%, respectively. The corresponding positive LRs for delivery < or = 34 weeks were 6.8, 3.8, 75.0, and 14.9, and within 14 days 9.7, 5.5, 107.3, and 17.1. The negative LRs were 0.6, 0.6, 0.7, and 0.3 and 0.5, 0.3, 0.6, and 0.2. They showed that the rapid phIGFBP-1-test has a high negative predictive value for preterm delivery, comparable to that of ultrasonographic cervical length measurement [35]. Paternoster et al. assessed phIGFBP-1 in cervical secretions and the sonographic measurement of cervical length in 210 symptomatic patients. They found that 26 mm was the best cut-off value for cervical length in terms of predicting preterm delivery (LR+, 3.69; LR−, 0.22), with a sensitivity of 86.4%, specificity of 71.9%, positive predictive value (PPV) of 34.5%, and negative predicting value (NPV) of 96.8%. They also found that the sensitivity, specificity, PPV, and NPV of phIGFBP-1 of a positive phIGFBP-1 test were 52.9%, 89.2%, 48.7%, and 90.8%, respectively, in predicting birth before 37 weeks' gestation with an OR of 9.3 (95% CI, 4.05–21.3), an LR+ of 4.9, and an LR− of 0.5 and that their combination had an NPV of 90%, greater specificity, and a better PPV (64.3%) than either method alone for preterm delivery [36]. Bittar et al. found that measuring cervical length at 22–24 weeks' gestation and phIGFBP-1 at 30 weeks' gestation improved the prediction of preterm delivery over either method used alone [37].

Audibert et al. reported that IGFBP-1 screening did not predict preterm delivery and fFN screening provided the best predictive capacity. A policy of contingent use of testing for fFN after CL measurement or contingent use of CL measurement after fFN screening (depending on available resources) is a promising approach to limit use of resources [38]. Cooley et al. studied the relationship between levels of insulin-like growth factors 1 and 2 (IGF-1, IGF-2) and insulin-like growth factor binding protein 3 (IGFBP-3) in antenatal maternal serum and gestational age at delivery. They reported that there was no significant association between maternal IGF-1 or IGF-2 and preterm birth (PTB). Maternal mean IGFBP-3 levels are significantly reduced in cases complicated by delivery <32 completed weeks [39].

Cervicovaginal beta-hCG measurement in patients with preterm labor may be used as a predictive test. Bagga et al. studied with a group of 100 women with a singleton pregnancy with preterm labour between 26–36 weeks' gestation. Cervicovaginal secretions were collected for HCG assay and cervical length was measured by transvaginal sonography (TVS). These parameters were analysed to predict preterm birth. The preterm delivery rate was 55%; 24% delivered within 48 h and 11% within 7 days of admission. The sensitivity, specificity, positive predictive value (PPV), and negative predictive values (NPV) of cervical length less than or equal to 2.5 cm to predict delivery within 48 h and 7 days of admission were 62.5%, 89.5%, 65.2%, and 88.3% and 60.0%, 96.9%, 91.3%, and 81.8%, respectively; and those of qualitative HCG were 87.5%, 80.3%, 58.3%, and 95.3% and 77.1%, 86.2%, 75%, and 87.5%, respectively. HCG value of > or = 45 mIU/mL was the optimal cut-off, with a sensitivity, specificity, PPV, and NPV for predicting delivery within 48 h and 7 days to be 95.8%, 73.7%, 53.5%, and 98.2% and 85.7%, 80%, 69.8%, and 91.2%, respectively. Combining either qualitative or quantitative HCG assay with cervical length significantly increased the sensitivity and NPV of cervical length alone for prediction of preterm delivery both within 48 h and 7 days. It was concluded that increased cervicovaginal HCG and reduced cervical length predicted an increased risk of preterm delivery in women with preterm labour. Qualitative cervicovaginal HCG assay may be used as a bedside test to predict preterm delivery within 48 h or within 7 days [40].

Adhikari et al. investigated prediction of the risk of preterm birth (<37 weeks) or early preterm birth (<34 weeks) by cervicovaginal HCG and cervical length measured between 24 and 28 weeks of gestation in asymptomatic women at high risk for preterm birth. They reported that to predict delivery <37 weeks, cervical length <2.95 cm had a sensitivity, specificity, positive predictive value (PPV), and negative predictive value (NPV) of 75%, 80.1%, 71.4%, and 90.7% respectively, and cervicovaginal HCG > 4.75 mIU/mL had a sensitivity, specificity, PPV, and NPV of 70%, 61.81%, 40%, and 85%, respectively. To predict delivery <34 weeks, cervical length <2.65 cm had a sensitivity, specificity, PPV, and NPV of 50%, 85.50%, 23.08%, and 95.16%, respectively; and cervicovaginal HCG > 14 mIU/mL had a sensitivity, specificity, PPV, and NPV of 83.3%, 85.5%, 33.3%, and 98.3%, respectively. Cervical length was superior to predict delivery <37 weeks, whereas HCG was superior to predict delivery <34 weeks. Their combination was superior to predict preterm birth both <37 weeks or <34 weeks, than either parameter used alone [41].

Combined marker evaluation could be used as a sensitive parameter for identifying women at risk of spontaneous preterm delivery but it is not possible to obtain biomarkers in most of the clinics. Therefore, the evaluation of the cervix with utrasoundography is important.

## 3. Cervical Assessment by Ultrasonography

Cervix can be evaluated by transabdominal, translabial, and transvaginal ultrasound (TVU). Each technique has its costs and benefits; however, a review of the current literature will show that the transvaginal method of cervical assessment is the most reliable. TVU is objective, reproducible, and acceptable to patients. At the transabdominal approach, the cervix may not be visualised in up to 50% of cases unless the bladder is full, but bladder filling significantly increases the length of the cervix. The transperineal route is limited by both the inconsistency in correlation between transvaginal and transperineal measurements and the inadequate visualisation of the cervix in up to 25% of cases. Cervical changes such as dilatation of the internal cervical os with funneling (beaking) of the membranes can be easily appreciated by TVU, but not by digital examination [42–44]. The ultrasound images were analyzed to assess changes in the cervix that are associated with spontaneous prematurity and to evaluate ultrasonography as an indicator of the risk of preterm delivery [45]. 

**Figure 1:**
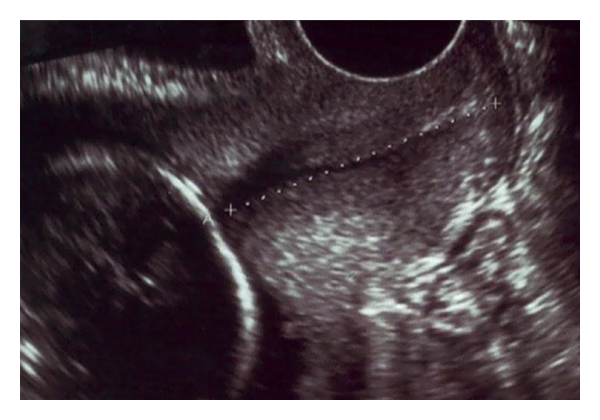
Transvaginal ultrasound image of the uterine cervix.

Before the evaluation of the cervix with transvaginal ultrasonography, first of all, the patient should have an empty bladder and be placed in dorsal lithotomy position. A distended bladder can alter the shape of the cervix and compass the cervical canal in some cases preventing the detection of cervical incompetence [44, 46]. The vaginal probe should be placed in the anterior fornix without pressure. If the probe is pressed too hard against the cervix, it can obscure cervical incompetence. Initial orientation is established by locating the sagittal view of the cervix. The cervical canal should appear as a hypoechoic groove. The junction between amniotic membrane and cervical canal is designated as the internal os. The external os is located at the lower end of the cervix. Cervical length (CL) is defined as the distance between the internal to external os along the endocervical canal (Figure 1). If the cervical canal is curved, the CL can be measured either as the sum of two straight lines that essentially follow the curve or by a straight line between internal and external os. A short CL is usually straight, and the presence of curved cervix generally signifies a CL greater than 25 mm and, therefore, is a reassuring finding [47, 48].

**Figure 2:**
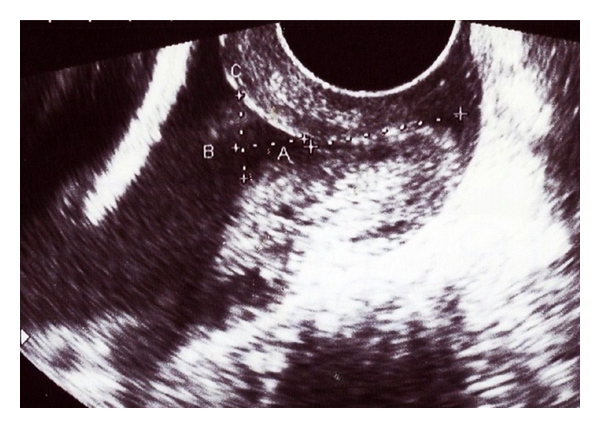
Transvaginal ultrasound image of the cervical funneling.

If the cervical canal is closed, CL is probably the only parameter that needs to be measured. If a normal appearing internal os cannot be visualized, the cervix should be assessed further to determine whether funneling (the internal os width is greater than 5 mm) is present (Figure 2). If funneling is present, the shape can be recorded [49, 50]. A continuous process of funneling has been described, going from a normal T shape to Y, then V, and finally a U shape. It appears that U shape is more likely to be associated with PTD, compared with a V-shaped funnel [51, 52]. 

CL during pregnancy can range from 25 to 70 mm and ultrasound width of the cervical canal ranges from 2 to 4 mm [47, 48, 53, 54]. Percentile values for CL between 17 and 32 weeks of gestation are indicated in Table 1 (unpublished data). Before 14 weeks, it is difficult to distinguish the lower uterine segment from the endocervical canal. Therefore, the measurement of the true cervical length is very difficult before 14 weeks. There is agreement that the best time to examine patients with this method to estimate their preterm birth risk is between 18 and 24 gestational weeks. Several studies reported that the measurement of cervical length in the first trimester is not predictive of preterm delivery [55, 56]. Finally, Greco et al. have recently reported that the endocervical length at 11 to 13 weeks is shorter in pregnancies resulting in spontaneous delivery before 34 weeks than in those delivering after 34 weeks [57].

Many parameters other than CL and presence or absence of a funnel have been studied including funnel width, funnel length, endocervical canal dilation, cervical index (funnel length +1/functional length), anterior and posterior cervical width, cervical angle, cervical canal contour, and cervical gland area (CGA) [58–60].

## 4. The Cervical Morphology and Biometry for the Prediction of Preterm Birth

The length of the cervix may be useful in predicting the risk of premature delivery, with a shorter cervix predicting a higher risk. A short CL is a better predictor of early PTD than later PTD [47, 61–63]. In a prospective multicenter study, Iams et al. performed TVS of the cervix in low-risk women with a singleton pregnancy at 24 weeks (*n* = 2915) and 28 weeks (*n* = 2531) of gestation. At 24 weeks, a cervical length of ≤25 mm had a sensitivity of 37%, a specificity of 92%, a positive predictive value 18%, and a negative predictive value %97 in predicting spontaneous preterm birth at <35 weeks' gestation. The RR of preterm birth before 35 weeks of gestation was about sixfold higher (95% CI: 3.84–9.97) among women whose cervical length was less than 25 mm than that among women with a cervical length above 40 mm [47]. 

To et al. conducted a population-based prospective multicentre study in 39 284 women with singleton pregnancies attending for routine hospital antenatal care in London, UK. The detection rate of spontaneous delivery before 32 weeks by measuring cervical length was 55%, with 10% false-positive rate [64]. Hibbard et al. measured the CL by TVS at 16–22 weeks in 760 singleton pregnancies in unselected women attending routine antenatal care. Relative risks (95% CI) for spontaneous preterm delivery before 37 weeks were 3.8 (2.6, 5.6), 5.4 (3.3, 9.0), and 6.3 (3.0, 13.0) for the tenth (30 mm), fifth (27 mm), and two and a half (22 mm) percentiles, respectively; RRs for before 35 weeks were 4.5 (2.9, 6.9), 7.5 (4.5, 12.5), and 7.8 (3.6, 16.7). Sensitivity ranged from 13 to 44%, specificity 90–99%, positive predictive value 15–47%, and negative predictive value 80–98% for prediction of preterm birth before 35 weeks [65]. 

A study of cervical length in low-risk women found an eightfold (95% CI 3–19) increased risk of preterm birth when the cervix was less than 30 mm at 18 to 22 weeks of gestation, but the sensitivity and positive predictive values were low: 19 and 6 percent, respectively [61]. Although low sensitivity and low positive predictive value limit its usefulness, it has high negative predictive values and it can be used in screening of low-risk obstetric populations (Table 2).

A number of studies have assessed the predictive value of TVS CL in women with some of the most important of these risk factors including a prior PTD [50, 66, 67], a history of excisional cervical procedures (cone biopsy, LEEP) [68, 69], mullerian anomaly [70], and two or more voluntary termination [70] (Table 3). In a prospective study of 705 high-risk women, the risk of spontaneous PTB before 35 weeks decreased by approximately 6% for each additional millimeter of CL (OR: 0.94, 95% CI: 0.92–0.95) and by approximately 5% for each additional week of pregnancy during which the CL was measured (OR: 0.95, 95% CI: 0.92–0.98). They conclude that gestational age at which transvaginal ultrasound cervical length is measured significantly affects the calculation of risk of spontaneous preterm birth. The spontaneous preterm birth risk increases as the length of the cervix declines and as the gestational age decreases [71]. 

Funneling comprising 40–50% of the total cervical length or a persistently shortened cervix (<25–30 mm) has, in several studies, been associated with an increased risk of preterm birth [17, 47, 49, 65]. To et al. measured cervical length among 6334 women with singleton pregnancies at 22–24 weeks and looked for the presence of funneling to evaluate its possible additional risk. Funneling of the internal os was present in about 4% of pregnancies and the prevalence decreased with increasing cervical length from 98% when the length was ≤15 mm to about 25% for lengths of 16–30 mm and less than 1% at lengths of >30 mm. The rate of preterm delivery was 6.9% in those with funneling compared to 0.7% in those without funneling. However, logistic regression analysis demonstrated that funneling did not provide a significant additional contribution to cervical length in the prediction of spontaneous delivery before 33 weeks (OR, for short cervix = 24.9 *P* < 0.0001; OR, for funneling = 1.8, *P* = 0.40) [50]. 

As an independent finding, funneling does not add appreciably to the risk of early gestational age at delivery associated with a shortened cervical length. So, women with a long cervix and funneling are not at increased risk of preterm delivery [50, 51, 72]. 

In the past few years publications have also highlighted the importance of another morphological ultrasonographic marker for PTD, named as the cervical gland area (CGA). The CGA is defined as the sonographically hipoechoic or hiperechoic zone surrounding the endocervical canal. If the CGA around the endocervical canal is not detected, it is defined as absent [58, 59]. 

Fukami et al. reported that the absence of CGA at second trimester ultrasonography appeared to be new and powerful predictor of PTD before 32 weeks gestation [58], similar to that reported by Pires et al. [59] (Table 4). Asakura et al. reported that short CL (<20 mm) with absent CGA represents an independent predictor for PTD. The absence of the CGA as a new marker for the risk of PTD has to be confirmed by further investigations [73]. 

## 5. Management Options for Short Cervix

Many interventions have been proposed in an attempt to prevent PTD in women at high risk.

Bed rest and hydration are often recommended in an attempt to prevent PTD in women at high risk, but there is no consistent evidence that they are able to delay delivery [74].

Progesterone's role in the treatment and prevention of preterm birth is still uncertain. A Cochrane meta-analysis from 2005 showed that intramuscular progesterone is associated with a reduction in risk of preterm birth before 37 gestational weeks [75]. Fonseca et al. in a multicenter, randomized trial proposed using daily vaginal micronized progesterone (200 mg) to women with CL 15 mm or less, irrespective of other risk factors. They showed a significant reduction in preterm birth at <34 weeks with intravaginal progesterone in patients treated based on premature cervical shortening as the indication for therapy [76]. O'Brien et al. analyzed 547 randomized patients with a history of preterm birth. They found that the progesterone-treated patients had significantly less cervical shortening than the placebo group. A significant difference was also observed between groups for categorical outcomes including the frequency of cervical length progression to ≤25 mm and a ≥50% reduction in cervical length from baseline in this subpopulation [67]. Further research is necessary and several randomized trials are underway to clarify the efficacy and fetal safety of progesterone treat.

Cervical cerclage is an old, easily performed procedure for treatment of true cervical incompetence [77]. Dijkstra et al. studied 80 women whose primary physician determined that a prophylactic (*n* = 50) or urgent cerclage (*n* = 30) was indicated and had transvaginal ultrasonographic evaluation before and after cerclage. They found that the mean ± standard deviation precerclage cervical length was 27.2 ± 10.3 mm and after cerclage was 34.1 ± 9.9 mm (*n* = 80, *P* < 0.001). The increase in cervical length after cerclage is not predictive of term delivery [78]. Until recently, cerclage was the only intervention studied to prevent PTB in asymptomatic women with short CL. Rust et al. randomized 113 women with CL < 25 mm or ≥25% funneling measured between 16 and 24 weeks to either modified bed rest or cerclage. No significant differences between two groups regarding risk of PTB <34 weeks or perinatal death were noted. It is important that women were included based on an incidental finding of a short cervix without taking into account other risk factors in the maternal history. Therefore, the majority of women were considered low risk before the sonographic findings [79]. To et al. also sampled 47,123 asymptomatic women and identified a cervix of 15 mm or less in 470, of whom 253 (54%) were randomized to either cervical cerclage (*n* = 127) or expectant management (*n* = 126). No significant differences in the rate of PTB <33 weeks or in perinatal or maternal morbidity or mortality were noted. Again, women in this study were incidentally found to have a short cervix. Subgroup analysis of the utility of cerclage in the high-risk population, based on maternal history, was not performed [80]. 

Cerclage is not indicated in low-risk patients. A case-controlled study by Incerti et al. found that cerclage does not reduce PTB in low-risk women with short CL compared with rest alone. However, this is not the case in women with previous PTD. However, this is not the case in women with previous PTD [81]. A recent multicenter randomized trial included 302 women with at least one prior PTB ≤32 weeks and TVU CL < 25 mm between 16 and 22^6/7^ weeks randomized to either cerclage or no cerclage. PTB <35 weeks was similar in both groups, but the benefit was most pronounced when CL was <15 mm, suggesting the presence of a more significant, and treatable, component of cervical insufficiency [82]. The systematic meta-analysis by Berghella et al. has shown that Cerclage, when performed in women with a singleton gestation, previous preterm birth, and cervical length <25 mm, seems to have a similar effect regardless of the degree of cervical shortening, including CL 16–24 mm, as well as CL ≤ 5.9 mm [83]. 

Weekly intramuscular 17-alpha-hydroxyprogesterone caproate (17 P) was compared with McDonald cerclage in women with short CL ≤ 25 mm at between 16 and 24 weeks' gestation. The study was terminated, however, when the interim analysis showed no difference in PTD <35 weeks between treatment groups. However, cerclage may be more effective in preventing spontaneous PTD in women with CL ≤ 15 mm [84]. In another study, Berghella et al. showed that 17 P had no additional benefit for prevention of PTD in women who had prior SPTD and got ultrasound-indicated cerclage for CL < 25 mm. In women who did not get cerclage, 17 P reduced previable birth and perinatal mortality [85].

 Evaluation of the cervical morphology and biometry with transvaginal ultrasonography at 16–24 weeks of gestation is a useful tool to predict the risk of preterm birth in low- and high-risk singleton pregnancies. For instance, a sonographic cervical length >30 mm and present CGA have a 96-97% negative predictive value for preterm delivery at <37 weeks. Transvaginal evaluation of cervix during routine fetal morphological examination helps identify asymptomatic low- and high-risk women for prediction of preterm delivery. Available evidence supports the use of progesterone to women with cervical length ≤25 mm, irrespective of other risk factors. In women with prior spontaneous PTD with asymptomatic cervical shortening (CL ≤ 25 mm) there should be prophylactic cerclage procedure and weekly to every two weeks follow-up.

## Figures and Tables

**Table 1:** Percentile values for CL between 17 and 32 weeks of gestation.

Group (GW)	Percentiles						
	5	10	25	50	75	90	95
17–20 GW	33,00	34,00	37,00	38,50	41,00	44,00	45,00
21–24 GW	29,00	30,00	34,50	37,00	39,00	41,00	43,00
25–28 GW	27,00	28,00	33,00	35,00	37,00	40,00	41,40
29–32 GW	26,50	28,00	31,00	33,00	36,50	39,00	40,00

**Table 2:** Studies of CL measured by transvaginal ultrasonography to predict preterm birth in low-risk women.

Authors	*n*	GW at testing	Outcome (GW)	Cutoff value (mm)	Sen. (%)	Spec. (%)	PPD (%)	NPD (%)
Tongsong et al. [6]	730	28–30	<37	<35	65.9	62.4	19.4	92.8

	2915	24	<35	≤30	54.0	76.3	9.3	97.4
Iams et al. [47]		24	<35	≤25	37.3	92.2	17.8	97.0
	2531	28	<35	≤30	69.9	68.5	7.0	98.5
		28	<35	≤25	49.4	86.8	11.3	98.0

Fukami et al. [58]	3030	16–19	22–31	≤30	50.0	98.5	8.3	99.9
			32–36	≤30	18.2	98.9	33.3	97.6

Pires et al. [59]	338	21–24	<37	<20	18.0	98.1	40.0	94.8
			<35	<20	27.3	97.9	30.0	97.6

Barber et al. [7]	2351	18–22	<37	<30	39.0	92.0	31.0	94.0

**Table 3:** Studies of CL in high-risk women with spontaneous PTD.

Authors	*n*	GW at testing	Outcome (GW)	Cutoff value (mm)	Sen. (%)	Spec. (%)	PPD (%)	NPD (%)	RR
Berghella et al. [43]	96	14–30	<35	25.0	59.0	85.0	45.0	91.0	4.8
Owen et al. [48]	183	16–24	<35	25.0	69.0	80.0	55.0	88.0	3.4
Crane and Hutchens [66]	193	24–30	<35	30.0	63.6	77.2	28.0	93.8	—
Adhikari et al. [41]	79	24–28	<37	29.5	75.0	80.1	71.4	90.7	—
			<34	26.5	50.0	85.5	23.1	95.2	—
Berghella et al. [68]	45	16–24	<35	25.0	60.0	69.0	35.0	86.0	2.5
Crane et al. [69]	75	24–30	<37	30.0	70.0	90.8	53.8	95.2	—
Airoldi et al. [70]	64	14–23^+6^	<35	25.0	71.0	91.0	50.0	95.0	13.5
Visintine et al. [72]	131	14–24	<35	25.0	53.0	75.0	48.0	78.0	2.2

**Table 4:** 

Authors	*n*	GW at testing	Test	Outcome (GW)	Sen. (%)	Spec. (%)	PPD (%)	NPD (%)
Fukami et al. [58]	3030	16–19	Absence CGA	22–31	75.0	99.8	54.5	99.9
			CL ≤ 30 mm + Absence CGA	22–31	50.0	99.8	40.0	99.9
			Absence CGA	32–36	2.3	99.7	18.2	97.2
			CL ≤ 30 mm + Absence CGA	32–36	2.3	99.7	20.0	97.2

Pires et al. [59]	338	21–24	Absence CGA	<35	54.5	99.1	66.7	98.5
			Absence CGA	<37	38.1	9.7	88.9	96.0
